# Neuralgia of the Scalp Cured by an Operation

**Published:** 1826-07

**Authors:** 


26. *Neuralgia of the Scalp cured by Operation.* A sailor, 40 years of age, was received into La Pitié, with a most excruciating complaint in his head, of which he gave the following account. Sometime previously he had been struck on the head by a squib or other fire-work at a public fête, which knocked him down, and burnt the scalp, on the left side of the lamdoidal suture, to the extent of half an inch. This, however, soon healed, and he thought no more of the matter. But, in the course of two months, he began to feel tinnitus in the left ear, wavering sight, and ultimately the most excruciating cephalalgia, the pain rising from the back part of the head and darting towards the left ear and the forehead. He went into an hospital for the complaint, and various means were tried to relieve him without success. At length he became so distressed with the pain that he determined on suicide. At this time he came under Lisfranc, in LA PITIÉ, who made an attentive examination of the part. When the old cicatrix was pressed, it caused considerable pain, and in this place it was observed that the lamdoidal suture sank in below the level of the surrounding parts, which suggested the idea, at first, that there had been fracture of the skull, with depression. The pain was always greater at night, and the unhappy man never got sleep. He had throbbing pain in the interior of the head, and his eyes would not bear the light. The treatment was commenced with general bleeding, twenty leeches to the part, low diet, &c. and the leeches were frequently repeated. But this plan produced no relief, and M. Lisfranc determined on an operation. An oval piece of scalp, including the seat of pain, three inches in length and two in breadth, was completely removed. Nothing particular could be detected in the vessels or nerves of the portion of scalp cut out. The pericranium underneath appeared sound. The effect was, that the neuralgia never appeared; and, as the man now lives at the hospital, as a nurse, it is ascertained that the cure is permanent.

